# Coil Embolization of Gastric Varices in Patient with Situs Ambiguus

**DOI:** 10.51894/001c.144549

**Published:** 2025-09-30

**Authors:** Yutong Liang

**Affiliations:** 1 College of Osteopathic Medicine Michigan State University

## 97

### BACKGROUND

Gastric varices (GV) pose a significant risk due to their association with high morbidity and mortality from bleeding. GV typically result from portal hypertension but may also occur due to vascular pathologies such as splenic vein thrombosis. Standard treatments include beta-blockers, and endoscopic interventions, while more invasive options like trans-jugular intrahepatic portosystemic shunt (TIPS) or balloon-occluded retrograde transvenous obliteration (BRTO) are reserved for refractory cases

This case presents a 45-year-old male with GV and situs ambiguus, a congenital condition where abdominal organs are irregularly arranged, which complicates diagnosis and treatment. Due to the absence of portal hypertension and gastro-renal shunt, TIPS and BRTO were unsuitable for treatment and trans-splenic coil embolization was successfully performed, with the patient being discharged in stable condition with no further bleeding episodes after follow-up.

### CASE PRESENTATION

Abdominal CT revealed situs ambiguus and an enlarged left-sided liver displaying Beaver tail anatomy, with a left-sided gallbladder and common bile duct, right-sided spleen and pancreas, and intestinal malrotation. Due to the patient’s unique anatomy, a trans-splenic approach allowed direct access to the splenic vein, enabling successful anterograde embolization of the gastric varices.

Ultrasound- guided trans-splenic access was first obtained. Embolization of the varices was performed using a combination of coils. Gelfoam pledgets were used for further embolization. Post- embolization venography did not opacify the submucosal gastric varices, demonstrating alternate vascular flow returning to the liver. Closure of the access was done by packing gel foam pledgets and “torpedoes” through the vascular sheath as it was retracted.

### DISCUSSION

Situs ambiguus poses a distinct diagnostic and therapeutic challenge, as organ arrangement is irregular, making interventions more complicated and requiring a customized approach. This case is unique due to the presentation of GV in a patient with situs ambiguus, a rare congenital anomaly. From literature review, a similar case involving situs inversus has been reported prior, in which BRTO and partial splenic embolization were performed. In conclusion, the successful management of this case demonstrates the importance of tailoring interventional strategies to each patient’s unique anatomy.

